# Detection of Acute Hepatitis C Outbreaks in California Using SaTScan: Statewide Spatiotemporal Analysis

**DOI:** 10.2196/104761

**Published:** 2026-10-07

**Authors:** Sarah New, Rachel McLean, Robert E Snyder, Beatriz Martínez-López

**Affiliations:** 1 California Department of Public Health, 850 Marina Bay Parkway, Richmond, CA, 94804, United States; 2 Center for Animal Disease Modeling and Surveillance, Department of Medicine & Epidemiology, Weill School of Veterinary Medicine, University of California, 2415A Tupper Hall, Davis, CA, 95616, United States, 1 530-752-1011

**Keywords:** hepatitis C, Space and Time Scan Statistics, SaTScan, space-time permutation, proof-of-concept, POC, outbreak detection, public health surveillance

## Abstract

**Background:**

Acute hepatitis C virus outbreaks in California are identified by local health jurisdictions through the investigation of cases reported by laboratories and health care providers to the state’s public health surveillance system. However, acute hepatitis C cases are widely underreported, limiting timely outbreak detection and early intervention. SaTScan (Space and Time Scan Statistics) has been proposed as a valuable tool to better detect disease clusters that may be missed by traditional surveillance, even when the disease is underreported, but it is not routinely used for acute hepatitis C surveillance. Timely outbreak detection enables prompt investigation, interruption of transmission networks, and expeditious access to treatment that prevents chronic hepatitis C progression.

**Objective:**

We assessed the feasibility of using SaTScan to identify verified acute hepatitis C outbreaks using public health surveillance data by conducting a retrospective cluster analysis followed by a prospective, proof-of-concept (POC) analysis that simulated routine surveillance at a single time point using the preceding 2 years of available data.

**Methods:**

We geocoded acute hepatitis C cases with an episode date between January 2022 and December 2023 that were reported to the California Department of Public Health (CDPH). Cases among people experiencing homelessness were included using proxy locations corresponding to their reporting jurisdiction. First, we applied a retrospective space-time permutation scan to determine if any detected significant clusters corresponded to a verified acute hepatitis C outbreak reported in California. We then performed a single prospective POC scan using surveillance data from August 2020 through August 2022 to simulate routine surveillance on August 28, 2022. Detection performance was evaluated based on whether the scan generated a signal corresponding to the known acute hepatitis C outbreak, measured using recurrence intervals.

**Results:**

Of the 236 acute hepatitis C cases reported in California with an episode date between January 2022 and December 2023, 97.9% (n=231) were successfully geocoded. The retrospective scan identified 1 significant cluster that corresponded to a verified outbreak in Los Angeles County. The prospective POC scan detected the same outbreak 1 day after the second outbreak-related case was reported, based on symptom onset. The cluster exceeded the recurrence interval threshold (1.7 y), and 2 of 3 outbreak-related cases were identified.

**Conclusions:**

SaTScan successfully identified a verified acute hepatitis C outbreak using both retrospective and prospective space-time permutation scans, demonstrating its potential to enhance real-time surveillance. However, detection performance depends on the timeliness and completeness of case reporting. Future research should explore the prospective use of SaTScan with real-time surveillance data to fine-tune signaling thresholds and scan statistic parameters and assess its broader applicability for acute hepatitis C outbreak detection.

## Introduction

Acute hepatitis C infection is defined as the 6-month period of disease onset following exposure to hepatitis C virus (HCV). Although patients with hepatitis C are often asymptomatic, symptoms after infection can include gastrointestinal issues (eg, abdominal pain, nausea, vomiting, dark-colored urine or clay-colored stools, and loss of appetite), fatigue, fever, and jaundice. Early diagnosis and treatment can prevent progression to chronic hepatitis C among the 55% to 85% of people with acute hepatitis C infection who do not spontaneously clear the virus [1-4]. Chronic hepatitis C left untreated can lead to additional complications, such as hepatocellular carcinoma and death [5]. Outbreaks of acute hepatitis C arise in settings where there is a high risk of percutaneous exposure to infected blood and where sharps are shared, such as among people who inject drugs (PWID) and in health care facilities. HCV is highly infectious and can remain viable on dried surfaces and equipment for up to 6 weeks, which increases the time frame for possible transmission and occurrence of outbreaks [6]. Poor injection safety practices in an outpatient clinic [7] and a contaminated multidose medication vial after the suspected reuse of a needle or syringe at a pain management clinic [8] have caused recent acute hepatitis C outbreaks in California. Persistent acute hepatitis C outbreaks occur more often among PWID and incarcerated individuals, where limited access to hepatitis C prevention interventions, such as sterile injection and tattooing equipment and medication for opioid use disorder, enable ongoing transmission [9-12].

In California, local health authorities identify acute hepatitis C outbreaks by investigating hepatitis C infections reported by medical providers or diagnostic laboratories. Standard case investigations are labor-intensive, requiring extensive reviews of patient medical records and laboratory results, confirmation of symptoms, exclusion of other hepatitis causes (eg, alcohol use, toxins, and certain medications), and in-depth interviews with both patients and their providers [13]. These steps are critical to public health surveillance, as they identify behavioral, health care, or cosmetic-associated risk factors and detect clusters of acute hepatitis C infections or point-source outbreaks. However, local health jurisdictions often struggle with competing priorities and limited staffing, reducing their capacity to rapidly identify cases of concern or those linked to outbreaks. These efforts are further complicated when patients are lost to follow-up or cannot be located. Acute hepatitis C infections may also be asymptomatic or present with nonspecific symptoms, contributing to underdiagnoses and underreporting [14]. National estimates suggest that each reported case of acute hepatitis C may represent up to 13.9 actual infections [15]. When outbreaks are identified early, local health jurisdictions can investigate potential transmission networks, notify contacts, and identify individuals who may be unaware of their exposure. A systematic method for detecting outbreaks is needed, even when exposure and risk information data are incomplete, to enable effective public health response, reduce transmission, and prevent progression to chronic hepatitis C and related sequelae.

SaTScan (Space and Time Scan Statistics; Martin Kulldorff and Information Management Services Inc) is a spatiotemporal analysis software that can detect disease clusters based on spatial and temporal patterns of reported infections, independent of available exposure information [16]. The New York City Health Department has used SaTScan for routine surveillance of 35 reportable conditions since 2014 [17,18], including to identify and promptly respond to outbreaks of both legionellosis [19,20] and salmonellosis [21]. Other jurisdictions nationwide have shown that SaTScan significantly reduced the time required to detect legionellosis by eliminating the need for time-intensive manual case review [22,23]. During the COVID-19 pandemic, many jurisdictions started using SaTScan for cluster detection to enable early intervention [24], aid with the allocation of resources [25], and highlight social inequalities [26]. SaTScan has also detected spatiotemporal clusters of conditions commonly co-occurring with hepatitis C, such as opioid overdoses [27] and HIV [28], helping to characterize neighborhood-level risk factors and predict areas where transmission was likely to occur. Despite its broad application in public health surveillance, SaTScan’s utility for acute hepatitis C remains underexplored.

We sought to test whether SaTScan could retrospectively identify outbreaks in noninstitutionalized, community settings across California among acute hepatitis C infections reported to the California Department of Public Health (CDPH). We also evaluated SaTScan’s potential use as an early warning system for acute hepatitis C by assessing the timeliness and precision of outbreak detection. To do so, we ran a single prospective scan on historical data restricted to acute hepatitis C infections that would have been available at the time an outbreak occurred, mirroring how SaTScan would have performed in real-time surveillance.

## Methods

### Data Source

Acute hepatitis C is a reportable infectious disease in California per Title 17, Sections 2500 and 2505 of the California Code of Regulations (CCR; [29]), with cases reported through the statewide surveillance system, California Reportable Disease Information Exchange (CalREDIE). Cases are reported to one of California’s 61 local health jurisdictions, comprising 58 counties and 3 city health jurisdictions (Berkeley, Long Beach, and Pasadena). However, for the purposes of this study, cases reported from city jurisdictions were counted in their respective county’s boundaries. Cases are identified either through seroconversion (ie, documented negative HCV antibody, RNA, or genotype test followed by a positive HCV result) or a clinical diagnosis by a health care provider who recognizes symptoms of acute infection. All case events reported in California, including among people with acute hepatitis C infections classified according to the national surveillance case definition [30] or those pending investigation, with an episode date between January 1, 2022, and December 31, 2023 were included in this analysis. We excluded cases reported from prison, jail, and federal facilities to focus on community transmission.

### Geocoding

We attempted to geocode all eligible acute hepatitis C cases reported to CDPH to obtain the latitude and longitude of the individual’s residential address. People experiencing homelessness (PEH) at the time of acute hepatitis C infection with no geocoded information were assigned to a synthetic location at the centroid of their reported jurisdiction and then shifted 10**°** west in longitude to position them outside populated areas. These displaced, centroid-based proxies served as artificial offshore “islands,” ensuring that cases without a known address did not artificially contribute to clusters in populated areas where the individual did not reside [18]. This approach enabled the retention of individuals experiencing homelessness, a population with increased prevalence of hepatitis C infection [31,32], in the spatial analysis, which would otherwise exclude them due to the absence of mappable residential address data.

### Criteria for Initiating Outbreak Investigations

Local health jurisdictions typically initiate outbreak investigations when 2 or more individuals with acute hepatitis C are identified in the same location (eg, the same health care facility, correctional setting, or other shared exposure site) within a 4-week period, when 1 individual with acute hepatitis C has no known risk factors (ie, sharing injection drug use or tattoo equipment, sexual contact with multiple partners, etc), or when 1 individual has health care–associated risk factors (ie, outpatient procedures, hospitalization, or organ transplantation).

### SaTScan Analysis

#### SaTScan Spatiotemporal Cluster Analysis

We used SaTScan (version 10.1) to detect spatiotemporal clusters of acute hepatitis C infections using circular scanning windows in both retrospective and prospective space-time scans [33]. The prospective scan was designated as a proof-of-concept (POC) analysis because it aimed to evaluate whether selected parameter settings could detect a known outbreak using only data available up to the second reported outbreak-related case. In both the retrospective and prospective scans, a space-time permutation model was selected because it does not require population at risk data for acute hepatitis C infection [34], which is difficult to estimate because of variations in drug use patterns, disparities in access to health care, and stigma. Multiple circular scanning windows in the permutation scan statistic varied by spatial width (geographical area) and temporal height (time period of interest), comparing the observed number of cases within each window to the expected number under the null hypothesis of no space-time clustering.

#### Retrospective Analysis/Parameter Selection

We conducted a retrospective space-time permutation scan on acute hepatitis C infections in California between January 2022 and December 2023. All acute hepatitis C cases classified as suspect, probable, or confirmed were included in the scan, regardless of the completeness of case investigations at the time of analysis. These inclusion criteria were intended to approximate case availability in real-time surveillance, when case classification may not yet be finalized. Initial SaTScan parameter settings were applied in the retrospective analysis to determine whether SaTScan could identify a known acute hepatitis C outbreak. Several parameters, including the space-time permutation model type, event types (eg, suspect, probable, or confirmed) for input data, maximum spatial cluster size, and secondary cluster reporting—were adopted directly from New York City Health Department guidance (Table 1) [18]. The maximum temporal cluster size, defined as the longest time period over which a cluster can occur, was set to 180 days to reflect the maximum incubation period for HCV (2‐26 wk) infection [14]. In SaTScan, the maximum temporal cluster size parameter limits the height of the scanning window, ensuring that clusters do not span longer than the specified time. The study period spanned 2 years, which is at least three times the maximum temporal cluster size, consistent with guidance indicating diminishing returns in statistical power beyond this threshold and improved baseline stability with a longer observation period [18]. Individual cases were aggregated into consecutive 7-day intervals, consistent with the temporal aggregation used in SaTScan, to reduce sparsity in daily case counts and improve the stability of the Poisson approximation. Cluster start and end dates therefore correspond to the boundaries of these 7-day intervals. The scanning window with the maximum likelihood was designated as the most likely cluster, that is, the cluster least likely to have arisen by chance [33]. Following New York City Health Department recommendations, we also explored overlapping clusters whose centers did not fall within any other cluster [18] because more than one acute hepatitis C outbreak could occur in California at any given time. Default settings were applied for additional parameters. Each acute hepatitis C case had a temporal analysis date defined as the episode date, which was the earliest among the case report date, date of symptom onset, date of diagnosis by a clinician, and date of specimen collection for diagnostic testing. Detected clusters with *P*<.05 were considered statistically significant.

**Table 1.** Space and Time Scan Statistics (SaTScan) parameter settings for a retrospective space-time permutation model to identify clusters of acute hepatitis C cases in California, 2022 to 2023.

Parameter	Setting	Justification
Study period	January 1, 2022, to December 31, 2023	Reflects the most recent surveillance data available and provides adequate observation time for stable background estimation
Events included^a^	Suspect, probable, and confirmed acute hepatitis C cases reported to CDPH^b^	All reported events were included to approximate case availability in real-time surveillance, when case classification may be incomplete
Model type^a^	Space-time permutation	Requires only case data; adjusts nonparametrically for purely spatial or temporal trends and geographical variation in the occurrence, diagnosis, or reporting of disease
Scan areas^a^	High rates	Detects areas with a higher-than-expected case count based on the space-time permutation likelihood compared to the null distribution
Time aggregation	7 days	Analysis run weekly
Maximum spatial cluster size (analysis)^a^	50% of all cases during the study period	Common practice to allow detection of both small and large clusters while minimizing assumptions about true cluster size
Maximum temporal cluster size	180 days	Maximum incubation period for acute hepatitis C infection
Maximum number of Monte Carlo replications^a^	999	Minimum number of replications required to avoid an unnecessary loss of power while balancing run time
Secondary cluster reporting criteria (output)^a^	No cluster centers in other clusters	Helps identify clusters in close proximity

a Adapted from published guidance from the New York City Health Department [18].

b CDPH: California Department of Public Health.

#### Prospective POC Analysis\Parameter Refinement

We conducted a prospective POC analysis to simulate routine surveillance at a single time point and refine parameters for prospective outbreak detection. The analysis used surveillance data from August 2020 through August 2022 to mimic routine surveillance on August 28, 2022, prior to recognition of the outbreak identified in the retrospective space-time permutation scan. The same parameter settings described in the retrospective analysis were initially applied to the POC prospective scan (Table 1) to evaluate their detection performance. Recurrence intervals (RIs) are used in prospective scans; a greater RI indicates a lower likelihood that the cluster is due to chance alone. We set an RI signal detection threshold of at least 100 days, meaning that when the null hypothesis of no clustering is true, the expected number of clusters with an RI of at least 100 days occurring by chance during any 100-day period is 1. This threshold aligns with New York City Health Department guidance, which classifies clusters with an RI from 100 days to less than 365 days as weak, 365 days less than 5 years as moderate, 5 years to less than 10 years as strong, and at least 10 years as very strong [18]. Because this was an exploratory scan, we prioritized sensitivity by including weaker signals that might still warrant follow-up under real-time surveillance conditions. For each detected cluster, we determined whether it corresponded to the verified acute hepatitis C outbreak and the proportion of cases within the cluster that were epidemiologically linked to the outbreak.

We then conducted a sensitivity analysis, iteratively evaluating alternative prospective parameter settings, to assess their effects on the detection of the known outbreak and the resulting RI. Parameters evaluated included maximum temporal cluster sizes of 30, 60, 90, and 180 days (analysis>advanced>temporal window), maximum reported spatial cluster sizes (output>advanced>spatial output), and daily vs weekly temporal aggregation (analysis). When temporal aggregation was varied, prospective analysis frequency (analysis>advanced>miscellaneous) was set to the corresponding interval and was not evaluated independently. Final settings were selected by balancing signal strength with epidemiologic plausibility and suitability for routine surveillance rather than by selecting the configuration that maximized RI.

### Ethical Considerations

This study was designed as a secondary analysis of original data collected to investigate hepatitis C infections as part of routine public health surveillance and is considered “not research” under the Common Rule (45 CFR [Code of Federal Regulations] 46; [35]). This determination was made by CDPH under its institutional policy for classifying routine public health surveillance activities conducted pursuant to the department’s statutory authority to investigate and control reportable diseases (CCR, Title 17, Sections 2500 and 2505 [29]); no formal institutional review board protocol application was submitted, consistent with CDPH policy that such surveillance activities do not constitute human subjects research.

## Results

### Retrospective Analysis

Between January 2022 and December 2023, 409 acute hepatitis C cases with an episode date during the study period were reported to CDPH. Of these, 173 (42.3%) cases reported from prisons, jails, or federal facilities were excluded. Of the remaining 236 reported acute hepatitis C cases, 231 (97.9%) were geocoded and included in the retrospective scan, including 31 (13.4%) cases among PEH. Among the remaining 5 (2.2%) acute hepatitis C cases with missing geocoded information, all were male participants, 4 (80%) were Hispanic, 2 (40%) resided in the state’s Central Coast region, and 3 (60%) resided in the Southern region of California. During 2022 and 2023, an average of 10 (SD 2.7) acute hepatitis C cases occurred each month (range: 4‐14), with no notable temporal trends. This reflects a relatively low number of cases during the study period. The local health jurisdictions with the highest number of reported acute hepatitis C cases were San Diego (n=120), Los Angeles (n=33), Fresno (n=15), and Orange (n=12) counties (Figure 1). The retrospective space-time permutation scan identified 1 significant (*P*<.05) cluster of 5 cases in Los Angeles County (Figure 2). All these cases resided within the same 12-mile radius, with episode dates that ranged from August 15, 2022, to September 18, 2022 (Table 2). Two of the 5 (40%) cases in this cluster were linked to an August 2022 outbreak reported in Los Angeles County.

**Figure 1.**
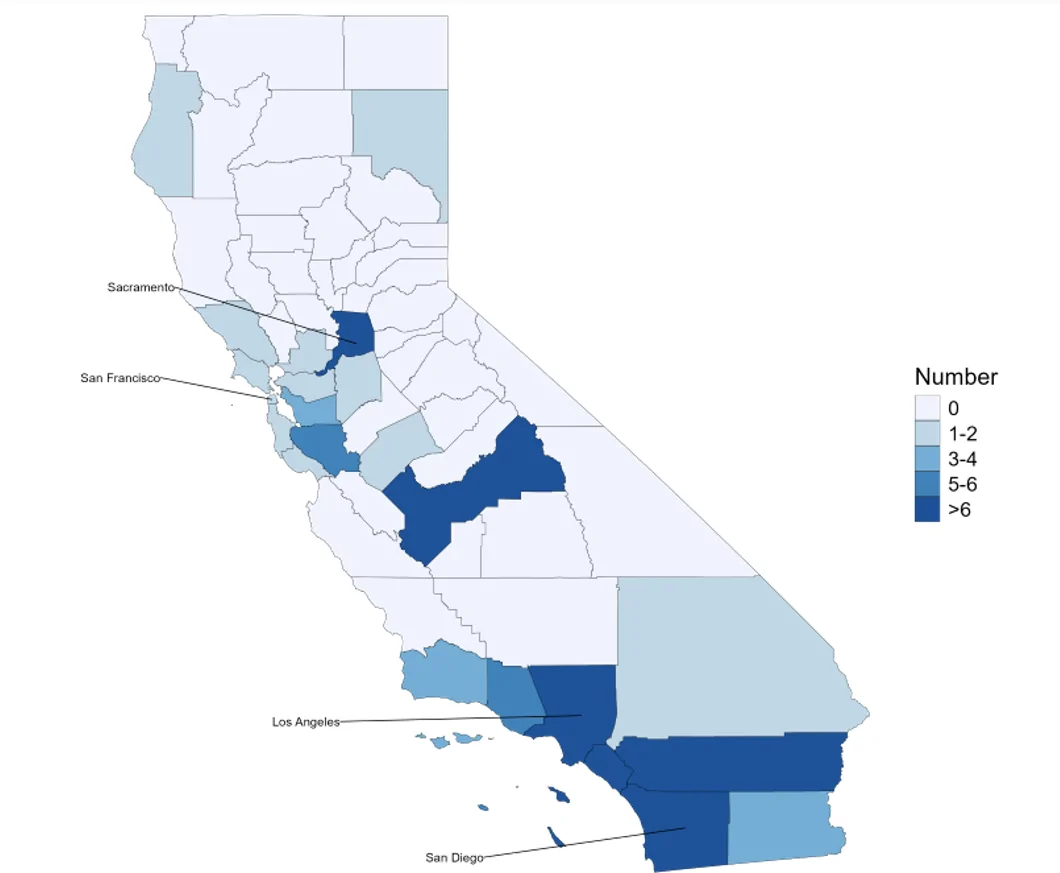
Number of acute hepatitis C cases reported to the California Department of Public Health (N=231) and included in the retrospective space-time permutation scan by local health jurisdiction, 2022 to 2023. All cases with geocoded information were included in the space-time retrospective scan. This includes cases among people experiencing homelessness (PEH) but missing a residential address, who were geocoded to their reported health jurisdictions. Shading indicates the number of reported acute hepatitis C cases by local health jurisdiction, with progressively darker shades of blue representing higher case counts (0, 1-2, 3-4, 5-6, and >6 cases).

**Figure 2.**
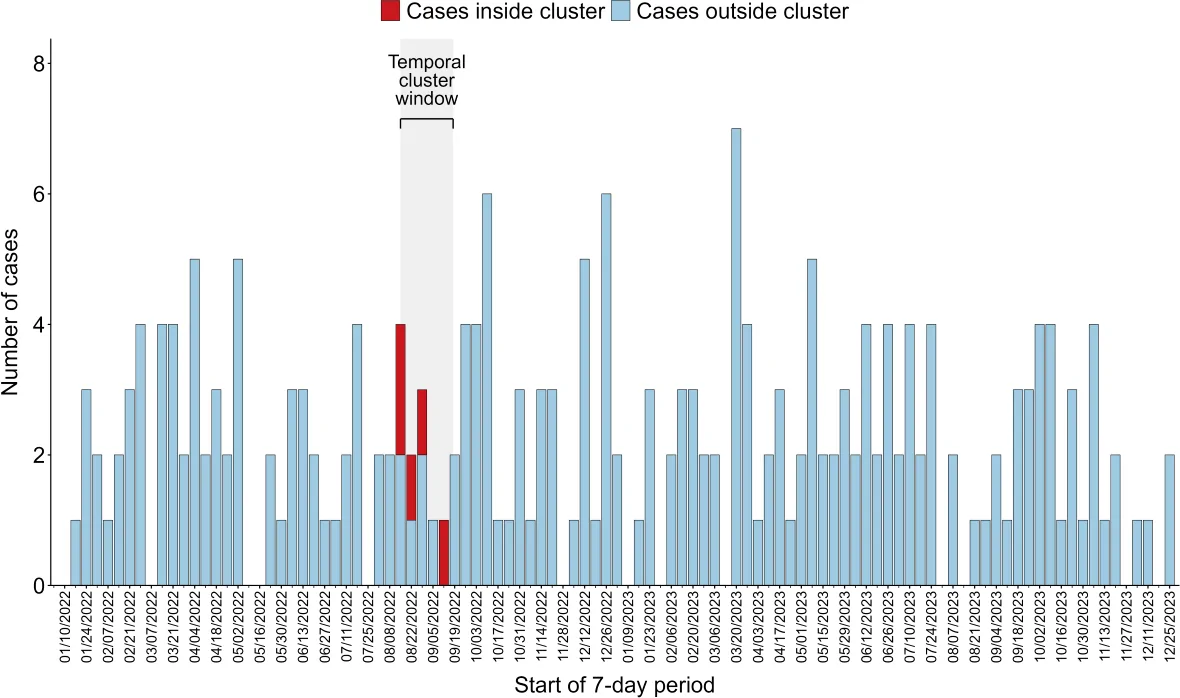
Weekly occurrence of acute hepatitis C cases (N=231) included in the retrospective space-time scan, California, 2022 to 2023. Cases with episode dates from January 1, 2022, through December 31, 2023, were aggregated into consecutive 7-day periods consistent with the temporal aggregation used in the SaTScan (Space and Scan Statistics) analysis.

**Table 2.** Significant cluster of acute hepatitis C cases in California detected by SaTScan (Space and Time Scan Statistics) using a retrospective space-time permutation model between January 1, 2022, and December 31, 2023.

Jurisdiction	Los Angeles County
Cluster time frame	August 15, 2022, to September 18, 2022
Cluster radius size, km	19.34
Observed cases	5
Expected cases	0.29
Log likelihood ratio	9.65
*P* value	.02

### Historical Cluster Summary

The retrospective scan identified a historical outbreak of acute hepatitis C transmission in August 2022 at a pain clinic in Los Angeles County [8]. The outbreak was suspected to be caused by contamination of a multidose medication vial, possibly due to the reuse of a needle or syringe [8]. Two acute hepatitis C cases with onsets 12 days apart in late August 2022 were linked through visits to the pain clinic on the same day. To our knowledge, this pain clinic outbreak was the only verified acute hepatitis C outbreak identified in a noninstitutionalized, community setting, including among PEH, in California during the study period. Following an exposure notification sent by the Los Angeles County Department of Public Health to 140 patients who had visited the pain clinic and were considered at risk, a third acute hepatitis C case was linked to the same exposure event [8]. This case was initially reported in another county; however, the patient moved out of state and was subsequently classified as out-of-state in the surveillance system. Because out-of-state cases were excluded from the data, this case was not included in this analysis.

### Prospective POC Analysis

To simulate how a prospective scan might have performed at the time the second outbreak-related case was reported, we geocoded 157 cases with episode dates between August 28, 2020, and August 28, 2022. The prospective space-time permutation scan detected 3 new acute hepatitis C clusters (Figure 3). One cluster matched the Los Angeles County outbreak previously identified in the retrospective scan, with a start date of August 28, 2020, and an end date of August 28, 2022 (Table 3). This cluster included 3 acute hepatitis C cases; 2 were known to be associated with the outbreak; and the third had no known link to the clinic exposure but had shared injection equipment with a non–outbreak-related acute hepatitis C case. The RI for this cluster was 1.7 years (approximately 620 d), a moderate signal, indicating that a cluster of this magnitude or larger would be unlikely to occur by chance more than once every 1.7 years. Because only 2 of the 3 cases were linked to the outbreak, the proportion of outbreak-related cases detected was 66.7%.

**Figure 3.**
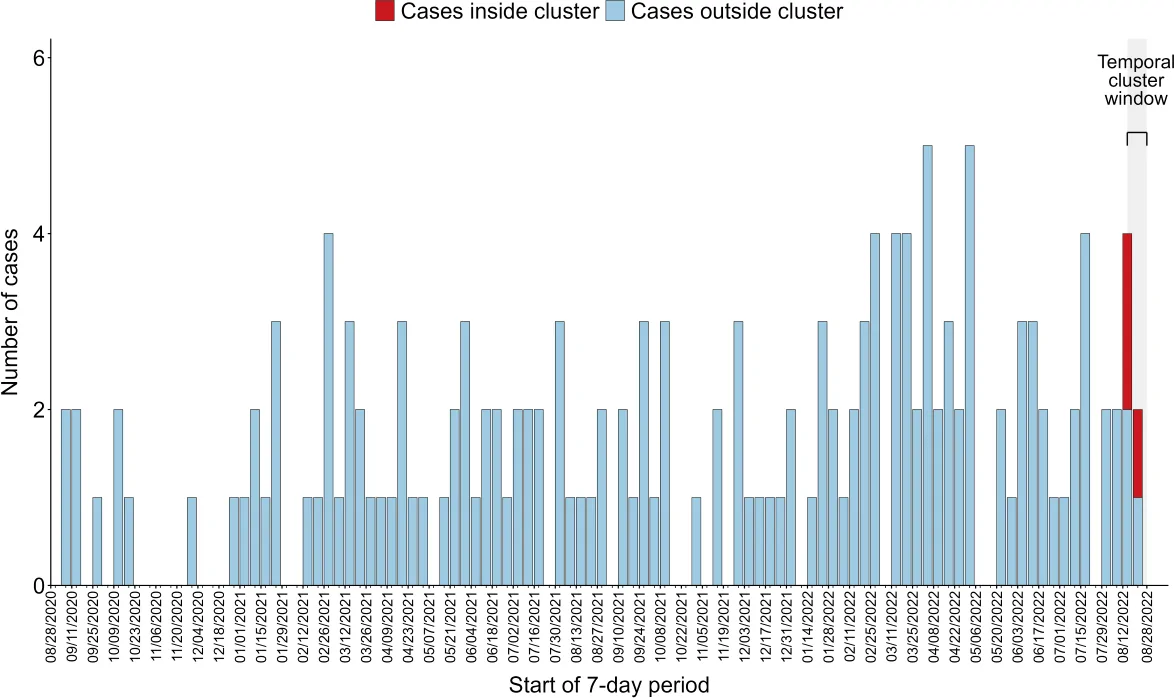
Weekly occurrence of acute hepatitis C cases (N=157) included in the prospective space-time scan, California, 2020 to 2022. Cases with episode dates from August 28, 2020, through August 28, 2022, were aggregated into consecutive 7-day periods consistent with the temporal aggregation used in the SaTScan (Space and Time Scan Statistics) analysis.

**Table 3.** Significant cluster of acute hepatitis C cases in California detected by SaTScan (Space and Time Scan Statistics) using a prospective space-time permutation model between August 28, 2020, and August 28, 2022.

Jurisdiction	Los Angeles County
Cluster time frame	August 15, 2022, to August 28, 2022
Cluster radius size, km	19.34
Observed cases	3
Expected cases	0.11
Log likelihood ratio	6.93
Recurrence interval, year	1.7

Sensitivity analyses demonstrated that shorter maximum temporal cluster windows generally produced equal or stronger RIs for the known outbreak. For example, 30-day and 60-day windows produced RIs of approximately 2 years compared with 1.7 years using the 90-day and 180-day windows. Temporal aggregation had a larger practical effect on signal detection; changing from weekly to daily aggregation reduced the RI from 1.7 years to 77 days, below the prespecified at least 100-day signal threshold. Restricting the maximum reported spatial radius to 10 km similarly reduced the RI to 32 days, whereas 20-km and 30-km settings retained an RI of 1.7 years.

## Discussion

### Principal Findings

Our findings demonstrate that SaTScan was able to detect a known acute hepatitis C outbreak in both the prospective and retrospective space-time permutation scans, suggesting potential utility for supplementing real-time surveillance. We used both retrospective and prospective space-time permutation scans to evaluate SaTScan’s utility in different contexts. Retrospective scans are useful for identifying historical outbreaks and informing initial parameter settings, while prospective scans can support routine monitoring for emerging clusters [36]. In the prospective POC analysis, the identified cluster met the predefined moderate RI threshold of at least 100 days and detected 2 outbreak-associated cases. Although 3 acute hepatitis C cases were ultimately linked to the outbreak, the third case was classified as out-of-state in the surveillance system and therefore excluded from the analysis. Case information and classification may have differed had the scan been conducted in real-time; therefore, whether this case would have been included or the resulting signal would have differed under routine prospective surveillance is unknown.

The outbreak-related cases identified involved patients from geographically distinct areas, illustrating how spatial methods may help identify related cases that do not share addresses or known epidemiologic links. Although Los Angeles County initiated an investigation through comparison of a pain clinic’s patient list to electronic lab reports, our prospective scan independently detected the same cluster using only routinely reported surveillance data. SaTScan identified the outbreak as a cluster of interest the day after the second outbreak-related case was reported, demonstrating how automated spatial methods could support near–real-time identification of clusters without requiring patient list matching to public health surveillance data. While Los Angeles County relied on a separate surveillance system during this study period and manually entered cases into CalREDIE, introducing reporting delays, this POC evaluation suggests that SaTScan could complement existing local workflows by systematically flagging clusters of concern when case ascertainment is timely. However, because only 1 verified community outbreak was available for evaluation, these findings demonstrate feasibility rather than the sensitivity or timeliness that could be expected across outbreaks more generally.

### Interpretation and Performance Considerations

Reported performance across previous SaTScan applications has varied, reflecting differences in disease epidemiology, case volume, and data quality. For legionellosis and salmonellosis, relatively moderate initial signals (eg, RIs of 1.4 y and 2.3 y) prompted targeted epidemiologic investigations and testing that ultimately confirmed point-source outbreaks [19,21]. Similarly, the present study identified a small, verified point-source outbreak with a moderate RI signal, demonstrating that epidemiologically meaningful outbreaks may produce relatively modest statistical signals. A simulation study of legionellosis found that an RI threshold of at least 100 days provided a balance between time to detection and positive predictive value, although a small, slowly developing simulated outbreak was not detected [22]. This limitation may also be relevant to acute hepatitis C, where small outbreaks developing gradually within populations with ongoing endemic transmission may produce less distinct spatiotemporal signals. Prospective legionellosis surveillance has also used an RI threshold of at least 100 days to identify clusters for further review [23]. Additionally, a COVID-19 application identified reporting delays and incomplete geocoding as limitations to the timeliness and completeness of prospective cluster detection [24]. These considerations are particularly relevant to acute hepatitis C, for which low case volume, incomplete ascertainment, and reporting delays may affect both the strength and timing of detectable signals. Other small acute hepatitis C outbreaks may also have occurred without being recognized or reported to CDPH and therefore could not be evaluated in this analysis. Together, these studies support the potential utility of prospective SaTScan for identifying signals warranting further investigation, while highlighting the importance of considering disease-specific epidemiology and surveillance data characteristics when selecting parameters and interpreting detected signals.

RI thresholds used to determine which clusters trigger alerts are suggested benchmarks and may be adapted based on epidemiologic and surveillance considerations [18]. Establishing useful thresholds may be particularly challenging when case volume is low because small clusters can be difficult to distinguish from expected variation [37,38]. Nevertheless, both space-time permutation analyses identified the known outbreak based on 2 eligible outbreak-associated cases, despite there being an average of only 10 (SD 2.7) acute hepatitis C cases reported statewide per month. Although this single outbreak cannot establish performance in other low-volume settings, it demonstrates that a small outbreak can generate a detectable signal even within sparse acute hepatitis C surveillance data.

While our findings suggest that SaTScan may have utility for detecting small, localized acute hepatitis C outbreaks, the approach has inherent constraints. Other cluster detection methods, such as flexible scan statistics [39], can identify irregularly shaped clusters and may capture more complex spatial patterns of transmission. However, these methods generally require greater computational and technical resources and are less commonly implemented in routine public health surveillance than SaTScan [40]. SaTScan itself also supports scanning along a user-defined network locations file, which can better accommodate irregularly shaped clusters than the circular scanning window used here; however, this approach requires aggregating cases to network nodes (eg, census tract centroids), introducing a trade-off between spatial flexibility and geographic precision. SaTScan has demonstrated comparable statistical power in many applications while requiring substantially less computation, making it a potentially practical tool for routine public health surveillance [41].

Sensitivity analyses demonstrated that parameter selection requires balancing statistical signal strength with epidemiologic plausibility and operational considerations. Although shorter temporal windows sometimes produced stronger signals, we retained the 180-day maximum temporal cluster size because it reflects the maximum incubation period for acute hepatitis C infection and allows detection of transmission occurring across the full biologically plausible interval. Similarly, restricting the spatial radius reduced signal strength, suggesting that overly narrow spatial windows may limit detection of epidemiologically related hepatitis C cases. This may be particularly relevant for point-source outbreaks, such as the outbreak detected in this study, because individuals sharing a common exposure may reside across a relatively broad geographic area. Hepatitis C transmission networks may also extend across geographically dispersed communities, particularly among PWID [42]. Thus, residential proximity does not necessarily reflect the geographic extent of a shared exposure or transmission network. Temporal aggregation also influenced signal strength, with weekly aggregation performing better than daily aggregation in these relatively sparse surveillance data. The space-time permutation model is based on a hypergeometric distribution conditioned on the observed spatial and temporal margins, but it currently uses a Poisson approximation to calculate the likelihood ratio [34]. The accuracy of this approximation may be affected when case counts are very low [43]. However, in our sensitivity analysis, changing from weekly to daily aggregation reduced the RI signal when case counts were sparse. The appropriate aggregation interval should therefore be considered alongside case volume and signal stability when configuring routine surveillance.

### Methodological Strengths

One difference in our study from previous analyses was the use of residential locations of cases, the smallest available geographic unit, rather than spatially aggregated data to census tracts or zip codes. Aggregating data can dilute spatial signals and expand the scanning window to include unrelated cases, decreasing cluster precision [43]. We initially attempted cluster detection at the census-tract level; however, no significant clusters were identified, consistent with signal dilution resulting from spatial aggregation. Technical guidance from SaTScan developers indicated that, given the sparsity of acute hepatitis C cases, case-level coordinates were appropriate for the goals of this study. Use of case-level residential addresses may also improve the timeliness of detection among localized clusters of smaller sizes [44]. Additionally, we applied an approach to retain acute hepatitis C cases among PEH, a population that is often excluded from place-based analyses due to missing address information. By assigning PEH to an artificial centroid [18], we were able to include 13.4% (31/231) of reported acute hepatitis C cases that otherwise would have been excluded. Retaining PEH is important because housing instability is associated with increased risk of hepatitis C infection, especially among PWID [45]. While clusters involving PEH were not verified as known outbreaks, excluding PEH, who may also have limited access to hepatitis C screening and are underrepresented in public health surveillance data, would further reduce completeness of case ascertainment and impede early outbreak detection for a population disproportionately affected by hepatitis C infection [46]. As a sensitivity analysis, we evaluated alternative approaches for assigning cases among PEH, including placing them at the county centroid rather than shifting coordinates outside the study area. Although no PEH cases were included in the known outbreak cluster, centroid placement altered the strength of the detected cluster, reducing the RI estimate. Assigning multiple cases without residential addresses to the same county centroid creates artificial spatial concentration among individuals who may not have been geographically proximate. In the space-time permutation model, this artificial concentration can alter the expected spatiotemporal distribution of cases and consequently influence the statistical strength of other clusters in the surrounding area. These findings support caution when assigning cases with unknown residential locations to a common geographic point. In future applications, assigning PEH based on the location of testing or diagnosis may provide a more meaningful alternative to either central placement or coordinate shifting.

### Limitations

This study has several limitations. First, only 1 verified community outbreak was available to evaluate the approach. Consequently, this study cannot estimate the sensitivity, specificity, or overall performance of SaTScan for acute hepatitis C outbreak detection. The findings instead provide preliminary evidence that this approach can detect a verified outbreak using routinely collected California surveillance data. Evaluation against additional verified outbreaks and prospective implementation are needed to characterize performance more broadly. Second, although our prospective POC analysis was designed to simulate real-time surveillance, it was conducted retrospectively using historical surveillance data. By the time of analysis, most case investigations had been completed and case classifications finalized, resulting in fewer pending cases than would likely be encountered in real-time surveillance. In practice, a higher proportion of cases would be pending investigation, some of which may ultimately be determined not to meet the acute hepatitis C case definition, potentially increasing background noise and generating false-positive signals [47]. Routine prospective implementation could address this by rerunning scans as investigations are completed and surveillance data are updated. Scans using a lagged end date could also identify clusters that were not initially detectable because of incomplete case classification or reporting [48]. Third, surveillance bias influences acute hepatitis C case ascertainment. Asymptomatic cases are less likely to be tested and reported during the acute phase, contributing to underreporting. Misclassification also occurs when providers test for suspected acute infections but do not submit the required acute case report to the health department, resulting in these infections being automatically classified as chronic through routine laboratory reporting. As a result, reported acute hepatitis C cases likely underestimate the true burden and may not accurately represent its spatial distribution, limiting the ability of any cluster detection method to identify outbreaks. Surveillance bias could be reduced by capturing negative HCV antibody and liver enzyme laboratory results in surveillance systems, increasing case ascertainment by identifying markers of acute infections, such as HCV antibody seroconversions and higher-than-normal alanine aminotransferase and bilirubin results. At last, this analysis excluded people who were incarcerated, who have a higher incidence of acute hepatitis C compared with people in community settings [49,50]. Because cases within carceral facilities are concentrated at the same geographic location, residential coordinates provide limited spatial information for detecting transmission patterns. Institutional settings may therefore be better evaluated using more granular spatial approaches, such as building-level analyses [51].

### Conclusions

This study suggests that SaTScan may be helpful for enhancing acute hepatitis C surveillance, particularly for identifying geographic clusters in settings where underreporting and incomplete data hinder early detection. We used a retrospective scan to evaluate initial parameter settings and a prospective POC scan to fine-tune parameters while simulating real-time surveillance using historical data. SaTScan detected 1 verified acute hepatitis C outbreak in both analyses, providing an initial POC framework that public health jurisdictions may adapt to refine parameter selection based on local surveillance practices, reporting patterns, and epidemiologic context. In practice, effective cluster detection depends on the timeliness and completeness of case reporting, as well as the granularity of the spatial information and the volume of case data available. Future research should evaluate SaTScan in real time to determine whether it consistently detects outbreaks early enough to support timely public health responses.

For implementation in California, SaTScan is planned to run automatically on a weekly basis, with epidemiology staff reviewing generated signals and communicating with local health jurisdictions when clusters exceed a predefined RI threshold of at least 100 days and warrant further investigation. Because scans can be automated, the primary ongoing staff effort is expected to involve the review and epidemiologic assessment of generated signals rather than manual execution of analyses. Beyond prospective implementation, validation against additional known outbreaks is needed to determine how well the approach performs across outbreaks with different spatial and temporal patterns, including more diffuse or network-associated transmission rather than a single point-source exposure. Other jurisdictions could use the parameters evaluated here as a starting point while adapting and validating them against their own surveillance data and epidemiologic context. Such evaluations will be important for determining the broader utility and generalizability of automated cluster detection for acute hepatitis C surveillance.

## Abbreviations

CalREDIE: California Reportable Disease Information Exchange
CCR: California Code of Regulations
CDPH: California Department of Public Health
HCV: hepatitis C virus
PEH: people experiencing homelessness
POC: proof-of-concept
PWID: people who inject drugs
RI: recurrence interval
SaTScan: Space and Time Scan Statistics
